# Supplementary material for: Cryo-EM structure of the agonist-bound Hsp90-XAP2-AHR cytosolic complex
Source: Nat Commun. 2022 Nov 16;13:7010. doi: 10.1038/s41467-022-34773-w (PMC9668932; doi:10.1038/s41467-022-34773-w)
Supplement: Supplementary file 1 — Supplementary Information [file 41467_2022_34773_MOESM1_ESM.pdf]

Supplementary Information

Cryo-EM structure of the agonist-bound Hsp90-XAP2-AHR cytosolic complex

Jakub Gruszczyk[1,*], Loïc Grandvuillemin[1], Josephine Lai-Kee-Him[1], Matteo Paloni[1], Christos G. Savva[2], Pierre Germain[1], Marina Grimaldi[3], Abdelhay Boulahtouf[3], Hok-Sau Kwong[1], Julien Bous[4], Aurélie Ancelin[1], Cherine Bechara[5,6], Alessandro Barducci[1], Patrick Balaguer[3], William Bourguet[1,*]

*Correspondence: jakub.gruszczyk@cbs.cnrs.fr, william.bourguet@cbs.cnrs.fr

Supplementary Figures 1-11 and legends

Supplementary Tables 1-4

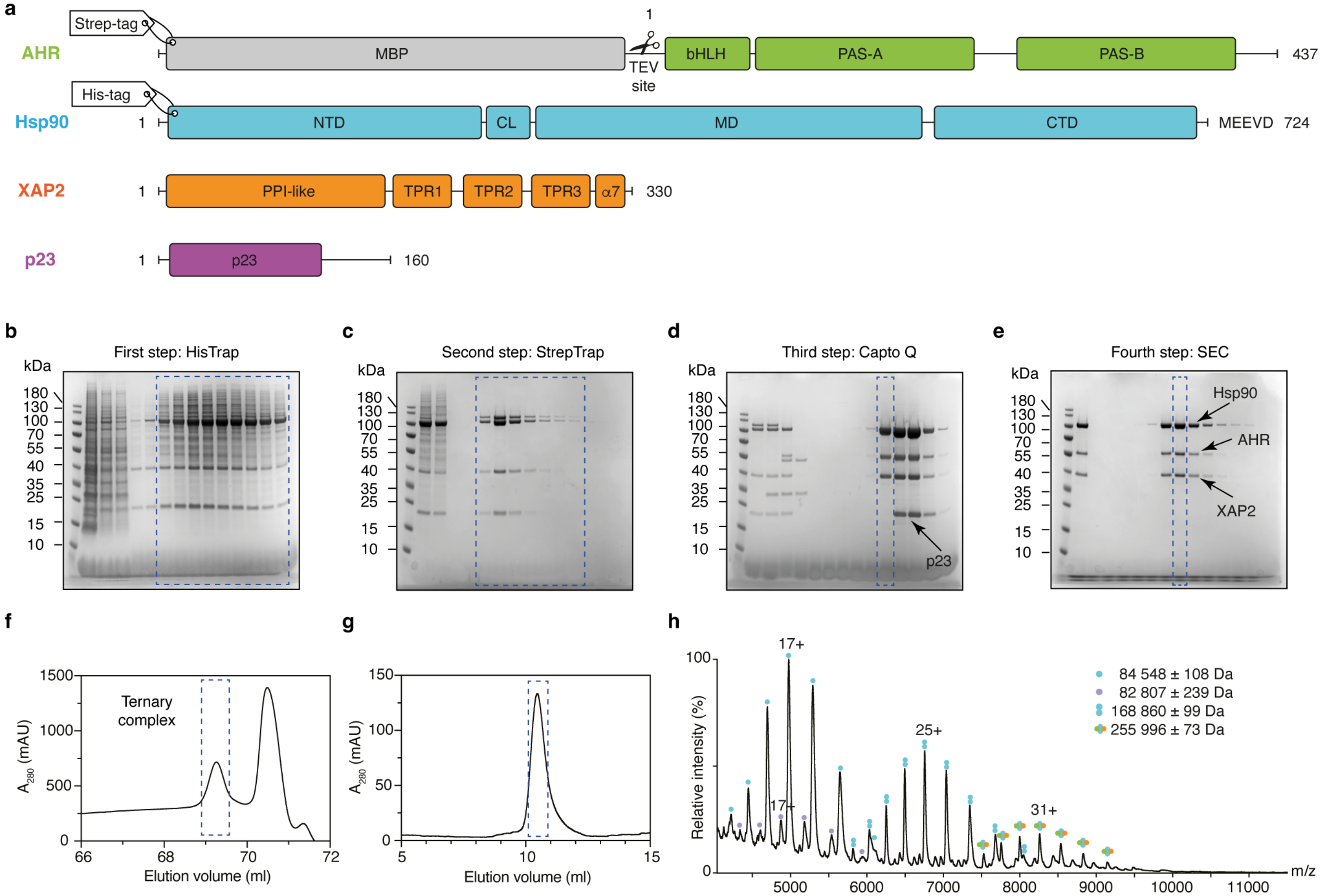

Supplementary Fig. 1 | Preparation of the Hsp90-XAP2-AHR complex. a, Schematic representation of the proteins used in this study. AHR construct includes N-terminal Strep-tag, followed by MBP protein and TEV protease cleavage site; bHLH - basic helix-loop-helix motif, PAS-A and PAS-B - Per-Arnt-Sim domain A and B. Hsp90 construct includes N-terminal His-tag; NTD - N-terminal domain, CL - charged flexible linker, MD - middle domain, CTD - C-terminal domain. XAP2 and p23 were left untagged; PPI-like - peptidyl-prolyl isomerase-like domain. TPR1, 2 and 3 - tetratricopeptide repeat motif 1, 2 and 3, α7 - C-terminal helix alpha. **b-e**, SDS-PAGE gels showing results obtained during particular steps of protein purification. Fractions used for the subsequent purification step are indicated with a blue dashed line. Molecular weight protein standards are indicated on the left-hand side of each gel. Gel images are representative of independent biological replicates (n=3). **f**, Elution profile from the ion exchange chromatography. A clear separation between ternary (no p23) and quaternary (with p23) complexes is visible; A_{280} - absorbance at 280 nm, AU - arbitrary unit. **g**, Size exclusion elution profile of the Hsp90-XAP2-AHR ternary complex displaying a monodisperse peak eluting at around 10.5 ml. **h**, Native MS spectrum of the protein sample at 1.8 mg/ml, showing the presence of both monomeric (84,548 ± 108 Da, single cyan circle) and dimeric (168,860 ± 99 Da, double cyan circles) Hsp90 species as well as the intact Hsp90-XAP2-AHR ternary complexes at 255,996 ± 73 Da. Some contaminant Sf9-derived Hsp90 is also present at low intensity (82,807 ± 239 Da, light purple circle); m/z - mass-to-charge ratio. All results from protein sample purification and MS experiments were successfully reproduced at least three times. Source data are provided as a Source Data file.

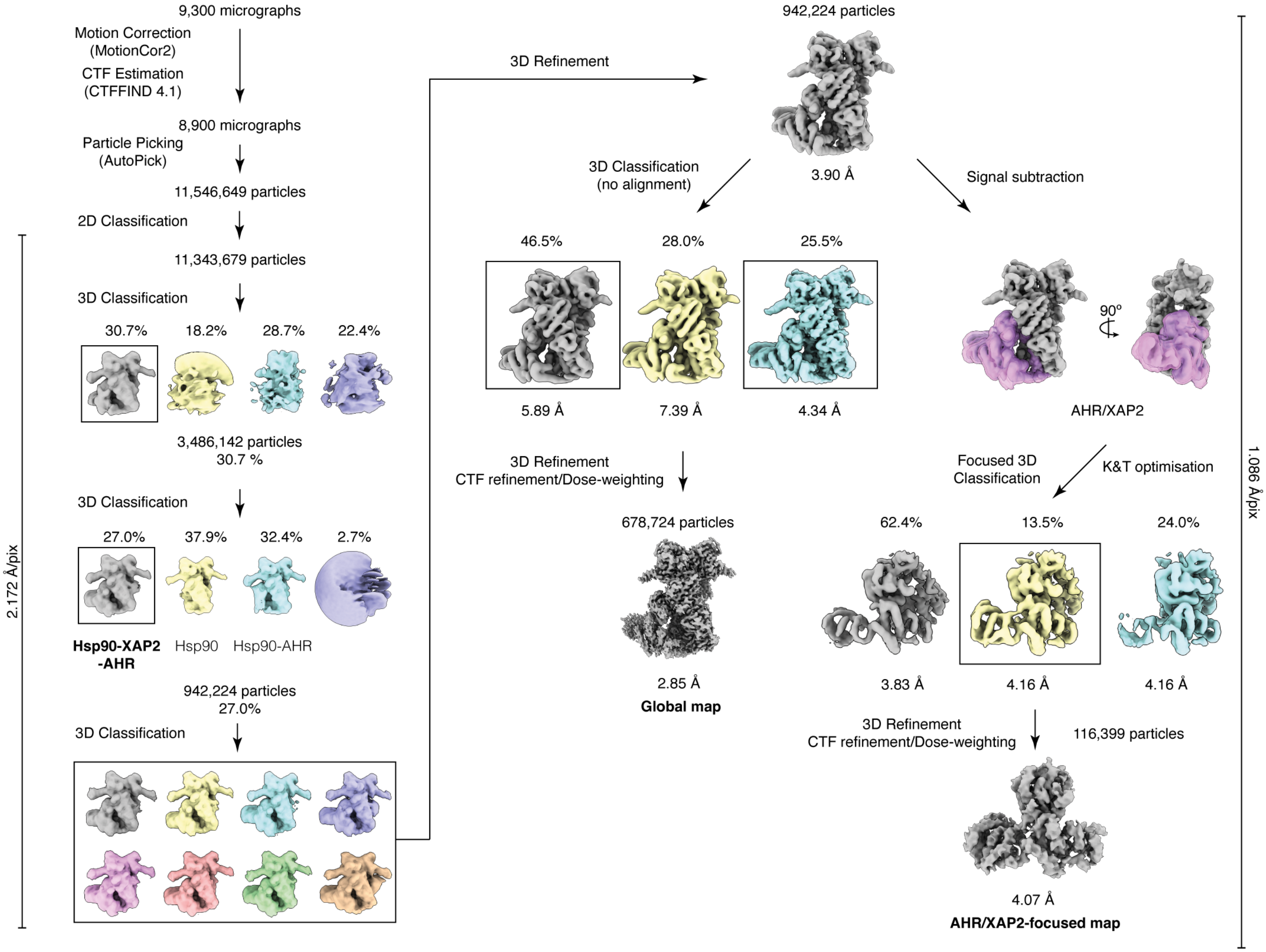

Supplementary Fig. 2 | Cryo-EM data processing workflow. A schematic representation of the data processing pipeline. For details, please see Materials and Methods section 'Cryo-EM data processing'. Soft mask used in focused 3D classification and refinement is shown as a semi-transparent surface and colored in purple.

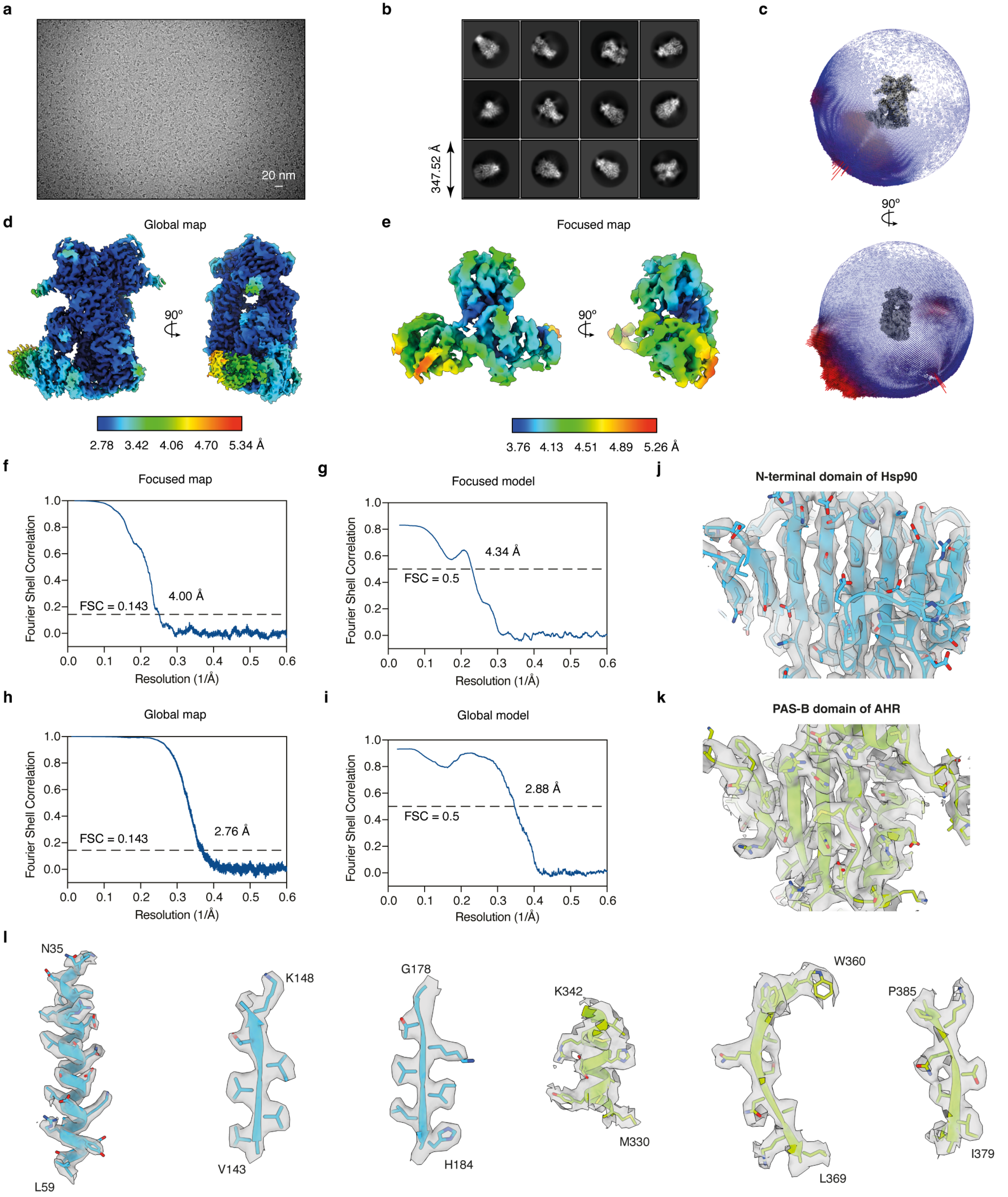

Supplementary Fig. 3 | Details of the cryo-EM data processing. a, Representative micrograph of the sample from 8,900 movies selected for particle picking. The scale bar represents 20 nm. **b**, Representative 2D class averages. 2D class averages exhibit different projections corresponding to each orientation. **c**, Euler angle distribution for the final reconstruction. The model is shown in two orthogonal views. **d**, Local resolution estimation diagram of the final map - global map. Resolution key is labelled from 2.78 to 5.34 Å. **e**, Local resolution estimation diagram of the final map - focused map. Resolution key is labelled from 3.76 to 5.26 Å. **f**, Fourier-shell-correlation (FSC) plot showing the resolutions at 0.143 FSC (drawn in a dashed line) determined by gold-standard method - focused map. **g**, FSC curve of the final refined model versus the final cryo-EM map - focused model. **h**, FSC plot showing the resolutions at 0.143 FSC (drawn in a dashed line) determined by gold-standard method - global map. **i**, FSC curve of the final refined model versus the final cryo-EM map - global model. **j**, Quality of the Hsp90-XAP2-AHR cryo-EM map: N-terminal domain of Hsp90. **k**, Quality of the Hsp90-XAP2-AHR cryo-EM map: PAS-B domain of AHR. **l**, Representative examples of the electron density map for chosen elements of secondary structure. Source data are provided as a Source Data file.

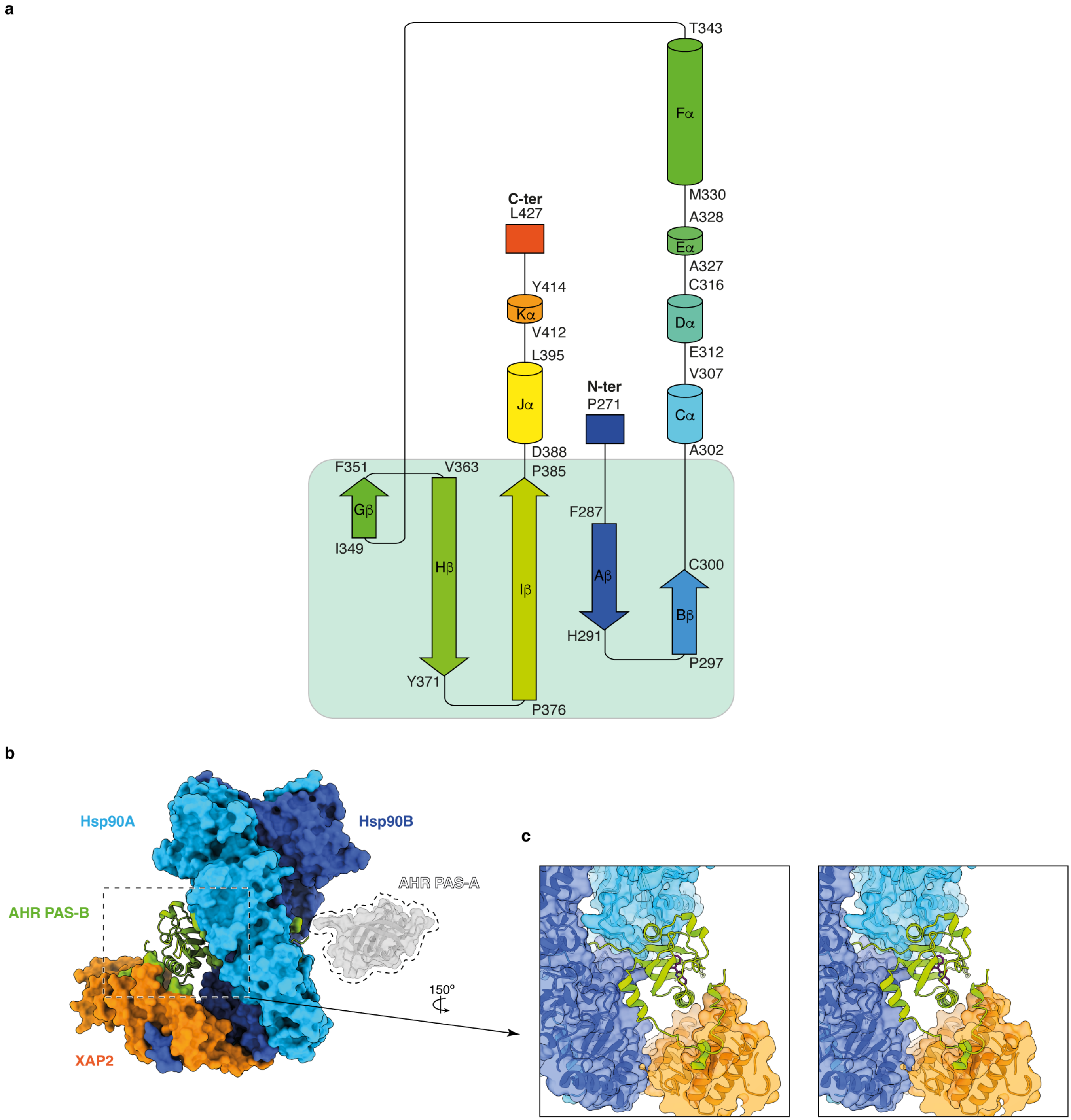

Supplementary Fig. 4 | Details of the organization of the AHR PAS-B domain. **a**, Schematic representation of the AHR PAS-B fold with indicated boundaries of the secondary structure elements. The five β-strands forming continuous β-sheet are highlighted in light green. **b**, Position of the PAS-B domain in respect to the other components of the complex. The location of the PAS-A domain (in grey) that could not be unambiguously resolved in the cryo-EM map is schematically indicated. **c**, Stereo view of the PAS-B domain and its interactions with the other components of the complex. The close-up view shows how PAS-B domain is closely surrounded by Hsp90 and XAP2. Indirubin is shown as magenta sticks.

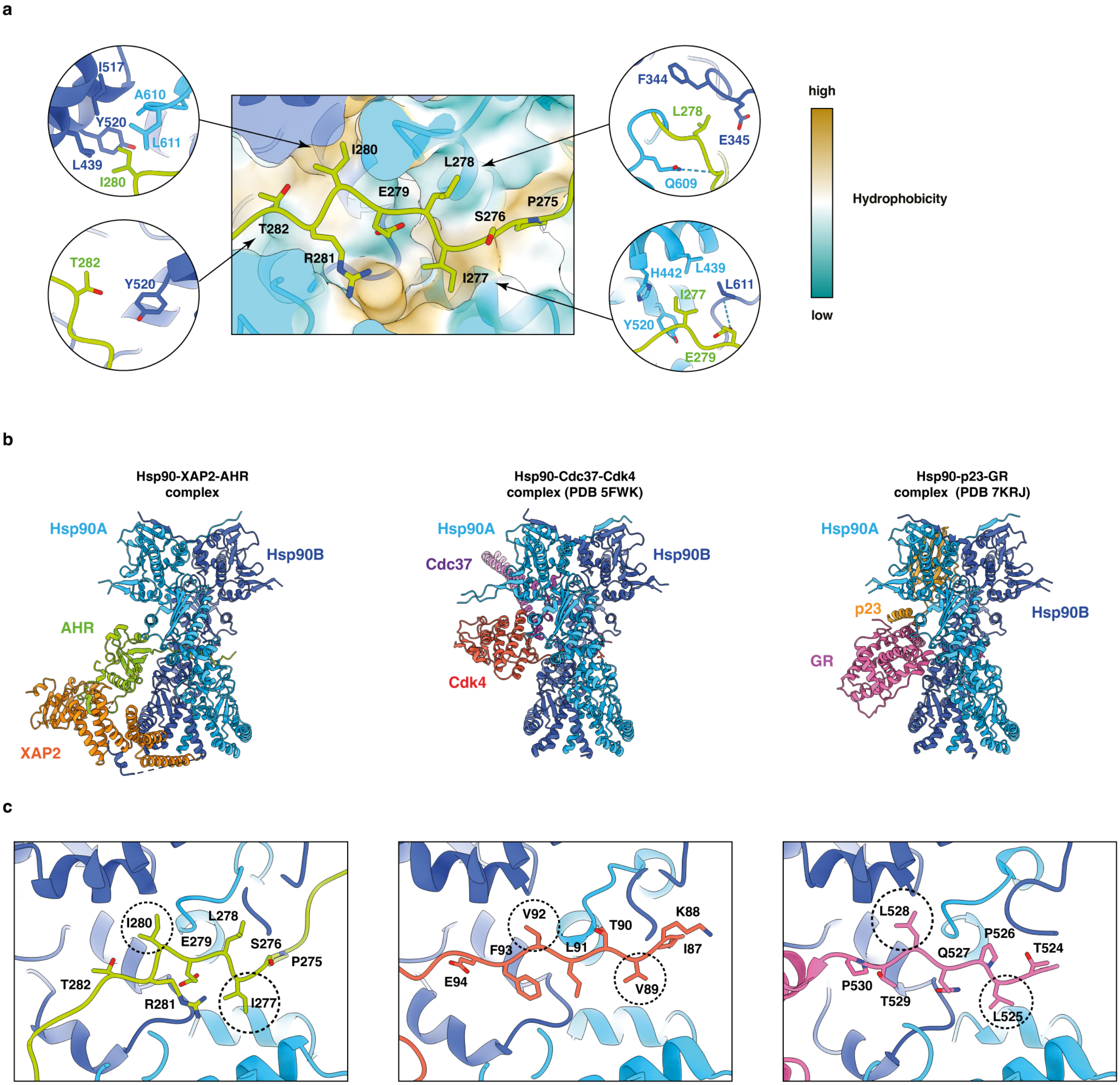

Supplementary Fig. 5 | Detailed analysis of the Hsp90 interactions with its client proteins. **a**, The AHR interdomain linker interacts with Hsp90. Two molecules of Hsp90 are shown in surface representation and colored according to the hydrophobicity of the residues. Detailed views of the interactions formed by the hydrophobic amino acid residues and T282 are shown in circles. Two isoleucine residues, I280 and I277, are facing towards hydrophobic patches located in both molecules of Hsp90. **b**, Comparison between three known structures of the Hsp90 client proteins: Hsp90-XAP2-AHR, Hsp90-Cdc37-Cdk4 (PDB 5FWK) and Hsp90-p23-GR (PDB 7KRJ) complexes are shown from left to right, respectively. Hsp90 adopts a closed conformation and two molecules of Hsp90 can be superimposed with the RMSD = 0.82 Å over 1,186 aligned atoms Cα for the complexes of AHR and Cdk4 and RMSD = 0.74 over 1,217 aligned atoms Cα for the complexes of AHR and GR. The client proteins and their corresponding co-chaperons are displayed in different colors. **c**, The close-up views of the superimposition of three structures focused on the AHR, Cdk4 and GR interaction with Hsp90. In all three structures the portion of client protein interacting with Hsp90 adopts a similar extended conformation. Residues involved in the interaction are shown in sticks and labelled. The conserved positions of the hydrophobic amino acid residues are indicated with dashed circles.

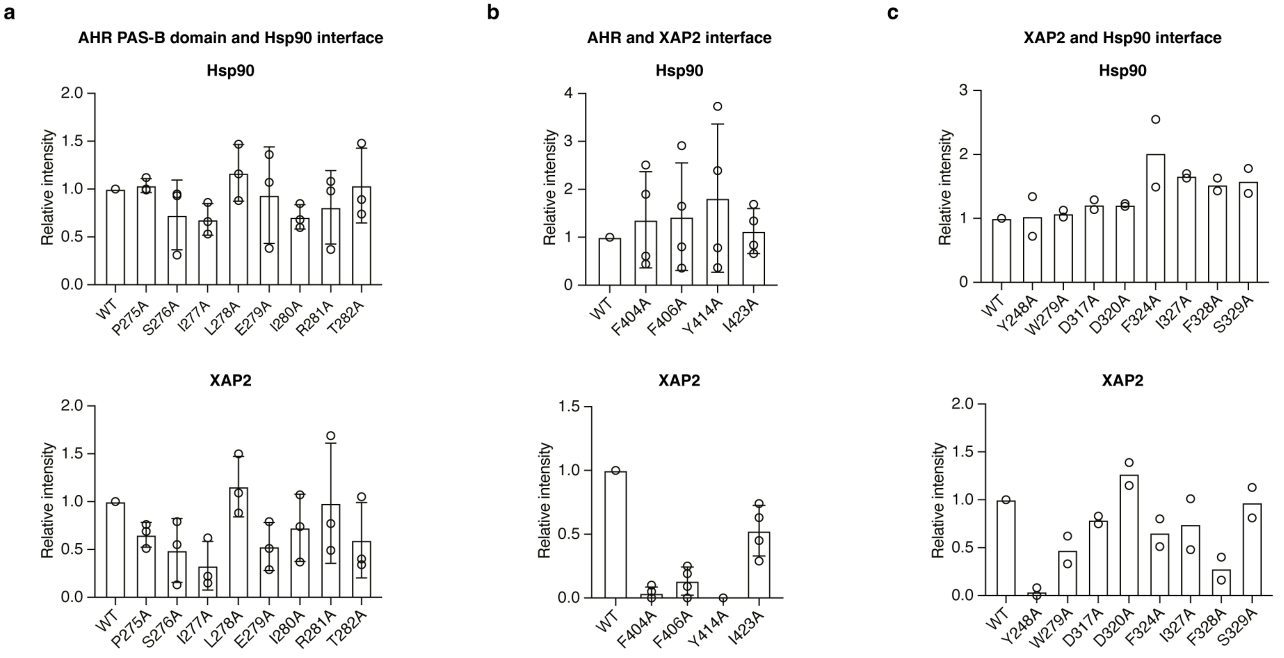

Supplementary Fig. 6 | Quantification of the immunoprecipitation experiments. In order to take into account different protein expression levels, the Western blot signal was normalised according to the level of immunoprecipitated AHR protein. The signal is expressed relative to the amount of wild type protein (100%). Top panel shows results for Hsp90 protein detected using anti-FLAG antibodies. Bottom panel shows results for XAP2 protein detected using anti-MYC antibodies. **a**, Results of Co-IP for the mutations within AHR linker. Bar graphs are presented as means ± standard deviation of biological replicates (n=3) **b**, Co-IP results for the AHR mutants at the interface with XAP2. Bar graphs are presented as means ± standard deviation of replicates (n=4) **c**, Co-IP results for the XAP2 mutants at the interface with Hsp90. Co-IP experiments were performed two times. Bar graphs represent mean value. Source data are provided as a Source Data file.

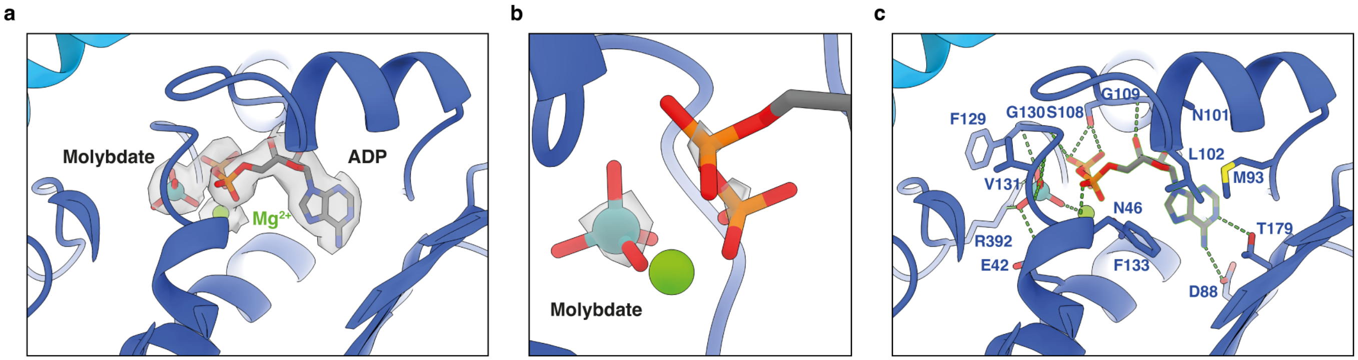

d

	Sample	Molybdate concentration (μM)
1	5 μM Na_2MoO_4	4.56
2	10 μM Na_2MoO_4	9.39
3	20 μM Na_2MoO_4	19.10

	Sample	Protein concentration (μM)	Measured molybdate concentration (μM)
1	Protein complex	8.3	8.43
2	Protein complex + AMP-PNP	7.1	0.83

Supplementary Fig. 7 | Detailed analysis of the nucleotide binding site of Hsp90. a, Close-up view of the nucleotide binding site in molecule B of Hsp90. The electron density map for the molecule of ADP, molybdate anion and magnesium cation has been displayed. **b**, Close-up view of the molybdate ion. Increased contour level of the map density shows a much stronger density relative to the α and β-phosphates. **c**, Details of the organization of the nucleotide binding site. Amino acid residues involved in the interactions are shown as sticks and labelled. Hydrogen bonds are indicated as dashed green lines. **d**, Left table: Results of the analytical mass spectrometry for three standard solution of sodium molybdate at 5, 10 and 20 μM, respectively. The measured values of the molybdate concentration in the samples are in good agreement with their theoretical values. Right table: Results of the analytical mass spectrometry for two samples of the protein complex. Sample #1 was prepared as described in 'Materials and Methods' section. The measured concentration of molybdate is similar to the protein concentration in the sample. Sample #2 was prepared the same way except before buffer exchange step the protein was incubated in the presence of 5 mM AMP-PNP for 1 hour. The measured molybdate concentration in the sample is low suggesting that the non-hydrolysable analog of ATP can displace molybdate from the nucleotide binding site. The results are representative of independent biological replicates (n=2).

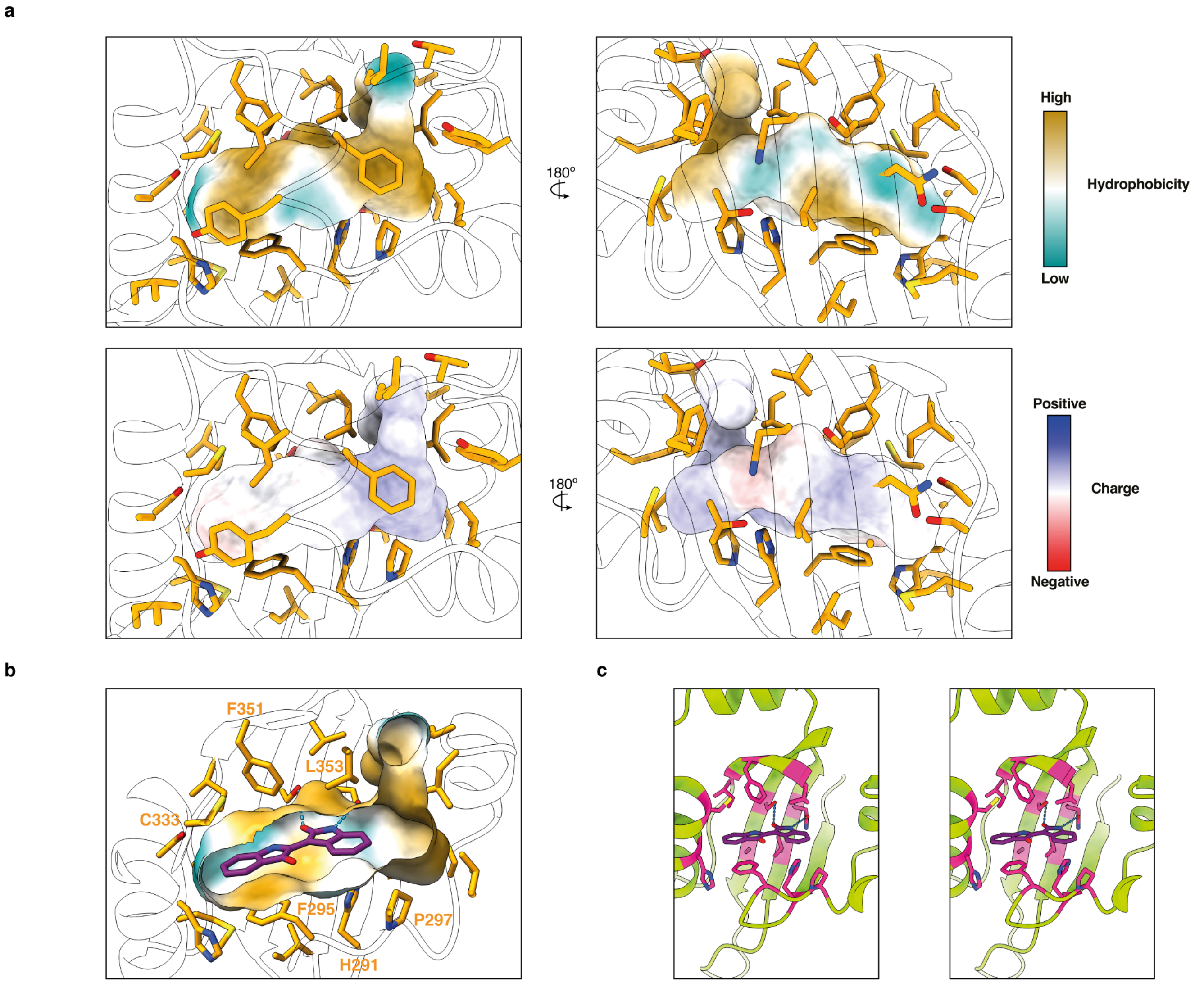

Supplementary Fig. 8 | Detailed analysis of the ligand binding domain of AHR. a, Amino acid residues involved in formation of ligand binding pocket (shown in sticks). The residues lining up the inside walls of the LBP are mostly hydrophobic. The surface of the LBP is colored either according to the hydrophobicity (top panel) or electrostatic potential (bottom panel). **b**, Close-up view of the interactions with indirubin. The ligand is occupying only a fraction of the LBP. Residues involved in constraining a planar conformation of the ligand are labelled. **c**, Stereo view of the ligand binding site.

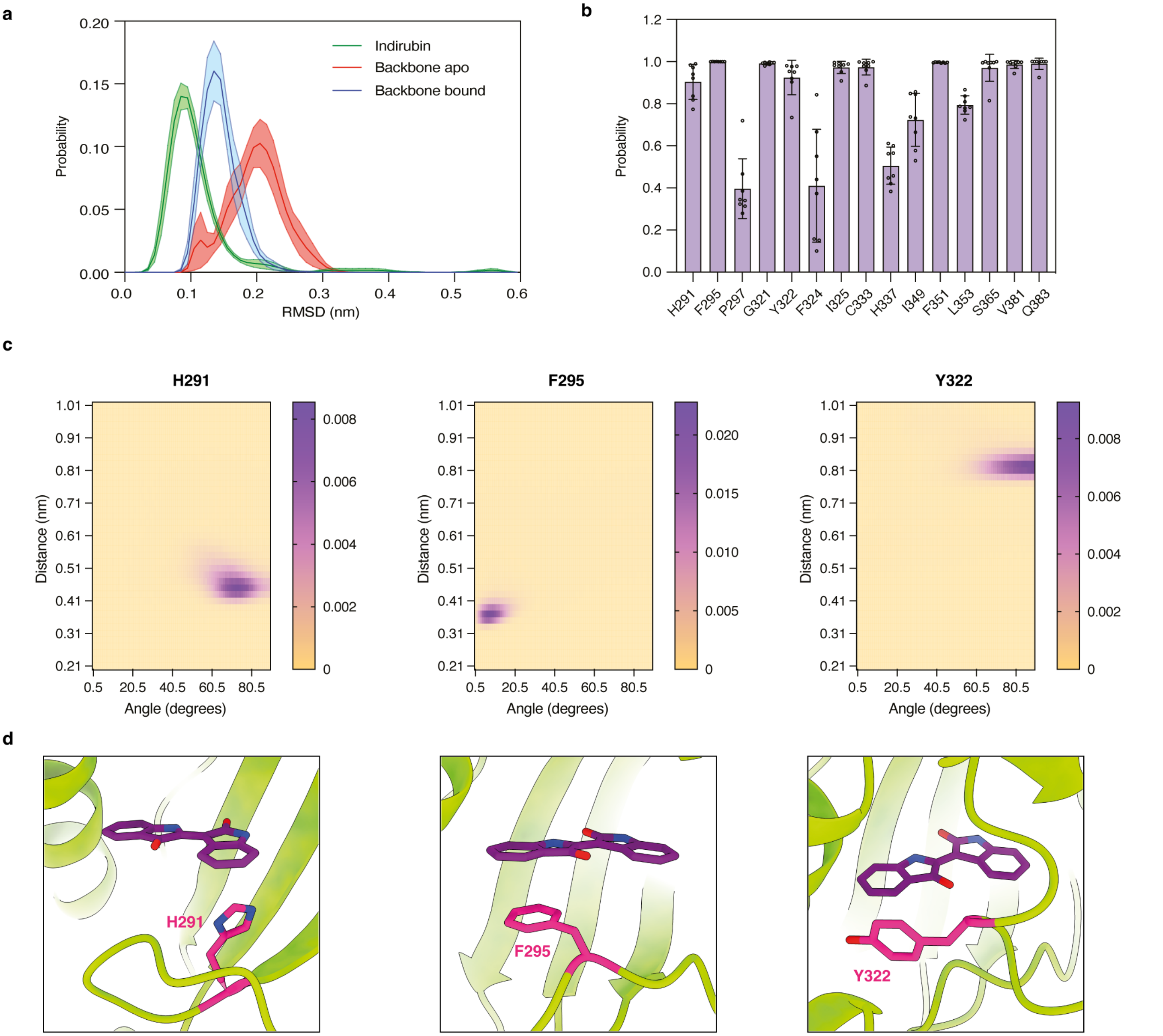

Supplementary Fig. 9 | Results of the Molecular dynamics simulation for PAS-B domain. a, Probability distribution functions (PDF) of the root mean square deviation (RMSD) of indirubin (green), and of the backbone atoms in the apo (blue) and bound (red) conformations. Values are averages over the MD simulations, while shaded areas are errors of the mean over the replicas (n=10 for apo system and n=8 for bound system). Indirubin remains remarkably stable in the experimental pose in the microsecond timescale, as indicated by an average RMSD with respect to the protein backbone of only 0.11 nm, with limited fluctuations of the position of the ligand. The protein conformation is destabilized by the absence of indirubin, as indicated by larger RMSD of the backbone for the apo system compared to that of the bound system. **b**, Contact probabilities between indirubin and the residues that form contacts with the ligand in the experimental structure. Data are presented as mean ± standard deviation of MD simulation replicas (n=8). Most of the contacts observed in the cryo-EM structure are remarkably stable in MD simulations in the microsecond timescale. Other contacts observed in the experimental structure are less stable, but these are formed with residues that are located at distances comparable with the cut-off value used for the definition of contacts. **c**, Two dimensional distributions of the distances and the angles between the aromatic rings of H291, F295, and Y322 with the indole rings of indirubin. (Left) H291 forms a stable T-shape interaction with an average distance between the centers of geometry of the rings of around 0.45 nm and angles between 60 and 80 degrees. (Center) F295, instead, forms a remarkably stable stacked π-π interaction with the indole ring of indirubin, with distances and angles between the two rings that are typical for this type of interaction. (Right) Lastly, Y322 and indirubin assume a T-shape conformation characterized by a rather long distance between the centers of geometry of the two rings, which is nonetheless stable in the microsecond timescale of the MD simulations. **d**, Close-up view of the interactions between residues H291, F295 and Y322 and indirubin. Source data are provided as a Source Data file.

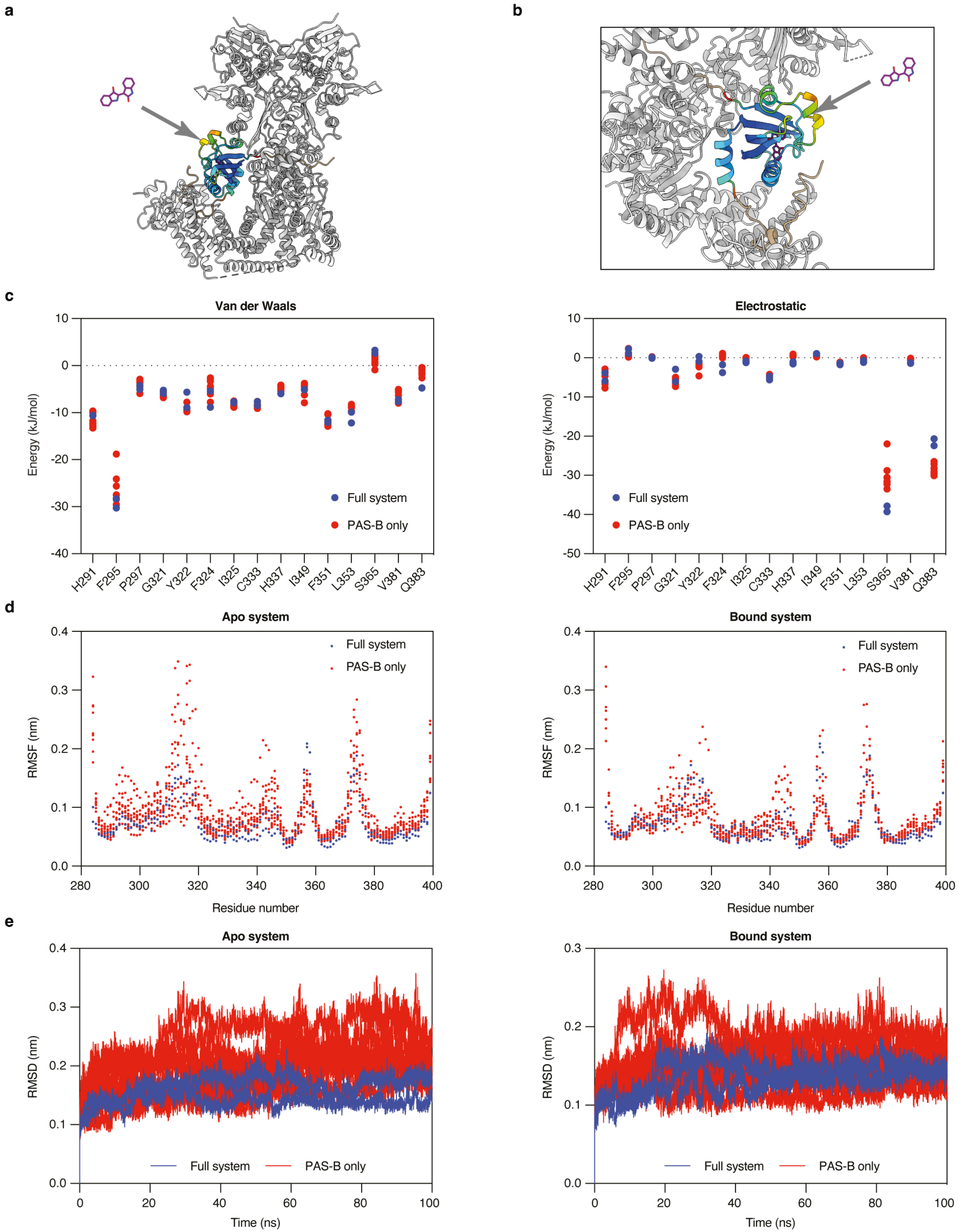

Supplementary Fig. 10 | Detailed analyses of ther ligand entry site. a, Localization of the entry site. The PAS-B domain is colored according to the RMSF. The other components are shown in grey The location of a potential ligand entry site to LBP is indicated with the arrow. The entry site is exposed to the environment and not occluded by any component of the complex. **b**, Close-up view of the entry site. **c**, Comparison of van der Waals (left panel) and electrostatic (right panel) interaction energies between indirubin and the residues of the PAS-B domain for the two MD simulations of the full complex (blue dots) and 8 MD simulations of the PAS-B domain (red dots). **d**, Comparison of the Root Mean Square Fluctuations (RMSF) of the backbone residues of the PAS-B domain between the MD simulations of the full complex (blue dots) and the first 100 ns of the MD simulations of the PAS-B domain alone (red dots) in apo (left panel) and indirubin-bound (right panel) simulations. **e**, Comparison of the timeseries of Root Mean Square Deviations (RMSD) of the backbone atoms of the PAS-B domain between the MD simulations of the full complex (blue lines) and those of the PAS-B domain alone (red lines) in apo (left panel) and indirubin-bound (right panel) simulations. Source data are provided as a Source Data file.

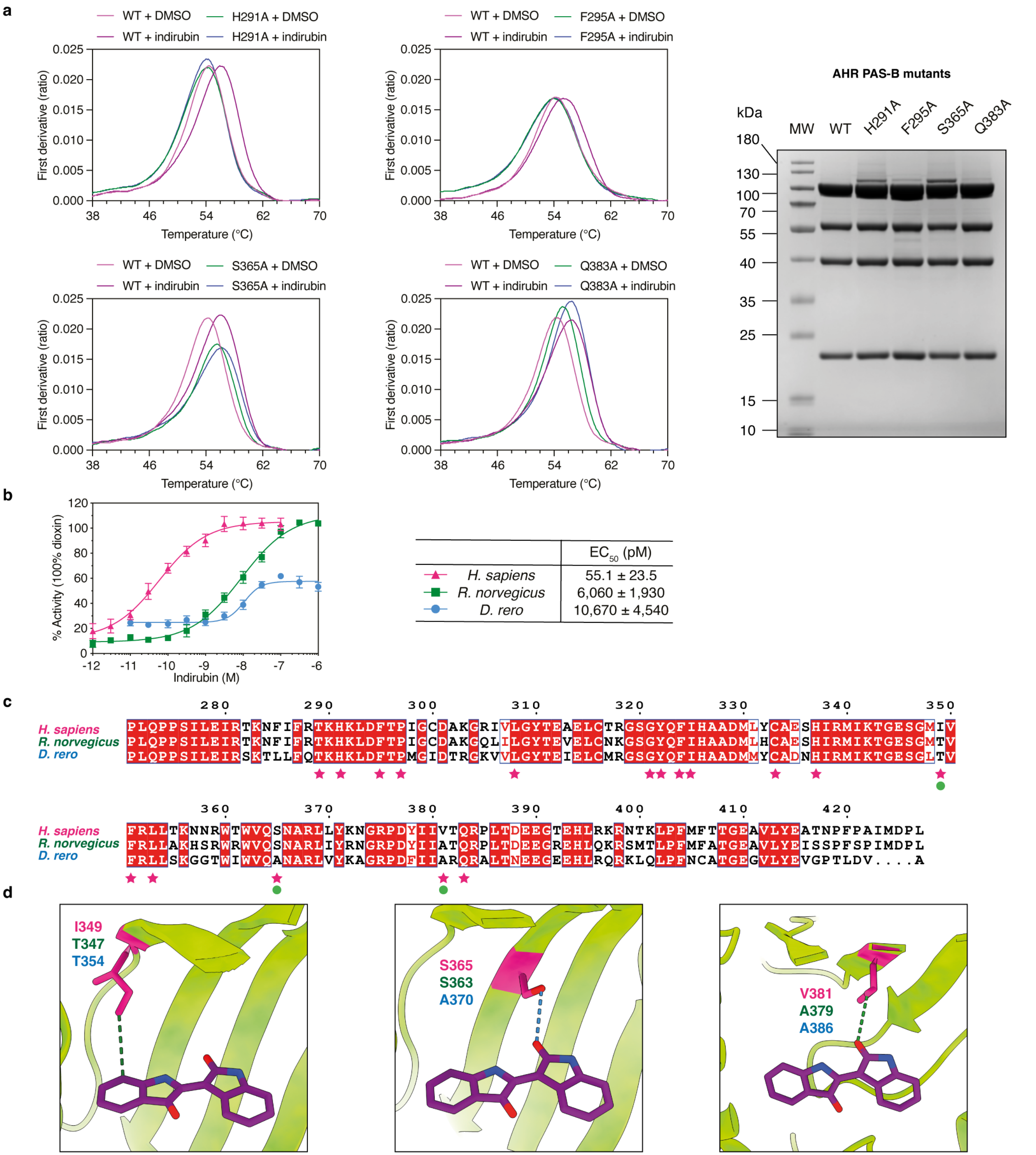

Supplementary Fig. 11 | Detailed analysis of the indirubin interaction with AHR. a, Nano-DSF analysis of the indirubin binding to the AHR PAS-B mutants. Mutations H291A, F295A and S365A render protein inactive in the indirubin binding assay. On the other hand, the Q383A mutation does not impair the interaction with indirubin. The first derivative of the ratio between intrinsic fluorescence at 350 nm and 330 nm is plotted against the temperature. The results are representative of independent biological replicates (n=3). The SDS PAGE gel shows the results of the purification for the prepared mutant proteins. The introduced mutations within PAS-B domain did not affect the formation of the complex. 10 μg of protein complex were loaded into each lane. The results are representative of independent biological replicates (n=2). **b**, Indirubin activity in transactivation assays using HAhLH (human), H4AhLH (rat) and ZAhLH (zebra fish) reporter cell lines. Indirubin is a much more potent activator of the human AHR compared with rat or zebra fish orthologs. The values of measured EC_{50} are indicated. For the human AHR, the same plot as in Figure 6b is shown. The results are represented as mean value ± SD from independent biological replicates (n=4). **c**, Sequence alignment of AHR sequence between three species corresponding to human residues 271-427. Residues that are conserved between all species are highlighted in red. Residues involved in the interactions with indirubin based on the cryo-EM structure are marked with magenta stars. Rat protein differs from human in 2 and zebra fish in 3 of those positions (marked additionally with green circles). **d**, Close-up view of the interactions formed by the residues I349, S365 and V381. Van der Waals interactions are indicated as dashed green lines. Hydrogen bonds are indicated as dashed blue lines. Source data are provided as a Source Data file.

Supplementary Table 1 | Cryo-EM data collection, refinement and validation statistics.

	**Hsp90-XAP2-AHR with indirubin** (EMDB-14971) (PDB 7ZUB)	**XAP2-AHR** (EMDB-14972)
**Data collection and Processing**		
Microscope	Titan Krios G3	
Voltage (kV)	300	
Camera	Gatan K3	
Magnification	81,000	
Pixel size (Å)	1.086	
Exposure rate on detector (e–/pix/sec)	15.0	
Exposure rate on specimen (e–/pix/sec)	16.5	
Number of frames	43	
Defocus range (μm)	-2.7 to -1.5	
Automation software	EPU	
Energy filter slit width (eV)	20	
Micrographs collected (no.)	9,300	
Total extracted particles (no.)	11,546,649	
**For each reconstruction**		
Final particle images (no.)	678,724	116,399
Point group	C1	C1
Map resolution (masked) (Å)	2.76	4.00
FSC threshold	0.143	0.143
Map resolution range (Å)	2.78-5.34	3.76-5.26
3DFSC Sphericity	0.965	0.838
Map sharpening B factor ($Å^2$)	-72.67	-192.52
Map sharpening methods	RELION3.1.1	RELION3.1.1
**Model Refinement**		
Initial model used (PDB code)	5FWP, 4F3L, 4ZPR, 2LKN, 4AIF	
Model resolution (Å)	2.88	
FSC threshold	0.5	
Model composition		
Non-hydrogen atoms	28,123	
Protein residues	1,722	
Ligands	2 MG, 2 MOO, 1 JY6, 2 ADP	
B factors ($Å^2$)		
Protein	64.56	
Ligand	32.57	
R.m.s. deviations		
Bond lengths (Å) (# > 4σ)	0.004 (0)	
Bond angles (°) (# > 4σ)	0.946 (1)	
Validation		
MolProbity score	5.44	
Clashscore	1.62	
Poor rotamers (%)	1.10	
Ramachandran plot		
Favored (%)	95.72	
Allowed (%)	4.28	
Disallowed (%)	0.00	

Supplementary Table 2 | Non-bonded interaction parameters for the molybdate ion atoms adapted from phosphate ion parameters.

Atom name	Atom type	σ (nm)	ε (kJ mol^{-1})	q (e)
P	p5	0.36940224	0.960228	0.828666
O1	o	0.30481209	0.6121192	-0.707000
O2	o	0.30481209	0.6121192	-0.707000
O3	o	0.30481209	0.6121192	-0.707000
O4	o	0.30481209	0.6121192	-0.707000

Supplementary Table 3 | Bonded interaction parameters for the molybdate ion atoms. Equilibrium bond length and angle amplitude have been taken from the experimental structure of the molybdate ions.

**Bond**	**Equilibrium bond length (nm)**	**Harmonic constant (kJ mol^{-1} nm^{-2})**
p5 - o	0.14870	443085.60
**Angle**	**Equilibrium angle amplitude (degrees)**	**Harmonic constant (kJ mol^{-1} rad^{-2})**
o - p5 - o	115.8000496	715.4640

Supplementary Table 4 | DNA sequences of the oligonucleotides used for the mutational studies.

No.	Primer	Sequence
1	AHR P275A, forward	ctgtttgcaattgccacaccgctgcaacctgcaagcattctggaaattcgtaccaaaaat
2	AHR P275A, reverse	atttttggtacgaatttccagaatgcttgcaggttgcagcggtgtggcaattgcaaacag
3	AHR S276A, forward	tttgcaattgccacaccgctgcaacctccggcaattctggaaattcgtaccaaaaatttc
4	AHR S276A, reverse	gaaatttttggtacgaatttccagaattgccggaggttgcagcggtgtggcaattgcaaa
5	AHR I277A, forward	gcaattgccacaccgctgcaacctccgagcgcactggaaattcgtaccaaaaatttcatc
6	AHR I277A, reverse	gatgaaatttttggtacgaatttccagtgcgctcggaggttgcagcggtgtggcaattgc
7	AHR L278A, forward	attgccacaccgctgcaacctccgagcattgcagaaattcgtaccaaaaatttcatcttc
8	AHR L278A, reverse	gaagatgaaatttttggtacgaatttctgcaatgctcggaggttgcagcggtgtggcaat
9	AHR E279A, forward	gccacaccgctgcaacctccgagcattctggcaattcgtaccaaaaatttcatcttccgc
10	AHR E279A, reverse	gcggaagatgaaatttttggtacgaattgccagaatgctcggaggttgcagcggtgtggc
11	AHR I280A, forward	acaccgctgcaacctccgagcattctggaagcacgtaccaaaaatttcatcttccgcacc
12	AHR I280A, reverse	ggtgcggaagatgaaatttttggtacgtgcttccagaatgctcggaggttgcagcggtgt
13	AHR R281A, forward	ccgctgcaacctccgagcattctggaaattgcaaccaaaaatttcatcttccgcaccaaa
14	AHR R281A, reverse	tttggtgcggaagatgaaatttttggttgcaatttccagaatgctcggaggttgcagcgg
15	AHR T282A, forward	ctgcaacctccgagcattctggaaattcgtgcaaaaaatttcatcttccgcaccaaacat
16	AHR T282A, reverse	atgtttggtgcggaagatgaaattttttgcacgaatttccagaatgctcggaggttgcag
17	AHR H291A, forward	cgtaccaaaaatttcatcttccgcaccaaagcaaaactggattttaccccgattggttgt
18	AHR H291A, reverse	acaaccaatcggggtaaaatccagttttgctttggtgcggaagatgaaatttttggtacg
19	AHR F295A, forward	atcttccgcaccaaacataaactggatgcaaccccgattggttgtgatgcaaaaggtcgt
20	AHR F295A, reverse	acgaccttttgcatcacaaccaatcggggttgcatccagtttatgtttggtgcggaagat
21	AHR S365A, forward	accaaaaataaccgttggacctgggttcaggcaaatgcacgtctgctgtataaaaacggt
22	AHR S365A, reverse	accgtttttatacagcagacgtgcatttgcctgaacccaggtccaacggttatttttggt
23	AHR Q383A, forward	aacggtcgtccggattacattattgttaccgcacgtccgctgaccgatgaagaaggcacc
24	AHR Q383A, reverse	ggtgccttcttcatcggtcagcggacgtgcggtaacaataatgtaatccggacgaccgtt
25	AHR F404A, forward	catctgcgtaaacgtaataccaaactgccggcaatgttcaccaccggtgaagcagttctg
26	AHR F404A, reverse	cagaactgcttcaccggtggtgaacattgccggcagtttggtattacgtttacgcagatg
27	AHR F406A, forward	cgtaaacgtaataccaaactgccgtttatggcaaccaccggtgaagcagttctgtatgaa
28	AHR F406A, reverse	ttcatacagaactgcttcaccggtggttgccataaacggcagtttggtattacgtttacg
29	AHR Y414A, forward	tttatgttcaccaccggtgaagcagttctggcagaagcaaccaatccgtttccggcaatt
30	AHR Y414A, reverse	aattgccggaaacggattggttgcttctgccagaactgcttcaccggtggtgaacataaa
31	AHR I423A, forward	ctgtatgaagcaaccaatccgtttccggcagcaatggacccgctgccgctgcgcaccaaa
32	AHR I423A, reverse	tttggtgcgcagcggcagcgggtccattgctgccggaaacggattggttgcttcatacag
33	XAP2 Y248A, forward	tgtcagtgtaaactggtggtggaagagtatgcagaagttctggatcattgtagcagcatc
34	XAP2 Y248A, reverse	gatgctgctacaatgatccagaacttctgcatactcttccaccaccagtttacactgaca
35	XAP2 W279A, forward	ttcaaacgcggtaaagcacatgcagcagttgcaaatgcacaagaagcacaggcagatttt
36	XAP2 W279A, reverse	aaaatctgcctgtgcttcttgtgcatttgcaactgctgcatgtgctttaccgcgtttgaa
37	XAP2 D317A, forward	caggcactggaagcacgtattcgtcagaaagcagaagaagataaagcccgttttcgcggt
38	XAP2 D317A, reverse	accgcgaaaacgggctttatcttcttctgctttctgacgaatacgtgcttccagtgcctg
39	XAP2 D320A, forward	gaagcacgtattcgtcagaaagatgaagaagcaaaagcccgttttcgcggtatttttagc
40	XAP2 D320A, reverse	gctaaaaataccgcgaaaacgggcttttgcttcttcatctttctgacgaatacgtgcttc
41	XAP2 F324A, forward	cgtcagaaagatgaagaagataaagcccgtgcacgcggtatttttagccattaactcgag
42	XAP2 F324A, reverse	ctcgagttaatggctaaaaataccgcgtgcacgggctttatcttcttcatctttctgacg
43	XAP2 I327A, forward	gatgaagaagataaagcccgttttcgcggtgcatttagccattaactcgagcatgcatct
44	XAP2 I327A, reverse	agatgcatgctcgagttaatggctaaatgcaccgcgaaaacgggctttatcttcttcatc
45	XAP2 F328A, forward	gaagaagataaagcccgttttcgcggtattgcaagccattaactcgagcatgcatctaga
46	XAP2 F328A, reverse	tctagatgcatgctcgagttaatggcttgcaataccgcgaaaacgggctttatcttcttc
47	XAP2 S329A, forward	gaagataaagcccgttttcgcggtatttttgcacattaactcgagcatgcatctagaggg
48	XAP2 S329A, reverse	ccctctagatgcatgctcgagttaatgtgcaaaaataccgcgaaaacgggctttatcttc
