# Supplementary material for: Cryo-EM structure of the agonist-bound Hsp90-XAP2-AHR cytosolic complex
Source: Nat Commun. 2022 Nov 16;13:7010. doi: 10.1038/s41467-022-34773-w (PMC9668932; doi:10.1038/s41467-022-34773-w)
Supplement: Supplementary file 3 — Description of Additional Supplementary Files [file 41467_2022_34773_MOESM3_ESM.pdf]

Description of Additional Supplementary Files

File name: Supplementary Movie 1

Description: Overall view of the agonist-bound cytosolic complex of AHR. The atomic model of the complex in shown in cartoon representation. The Hsp90A is coloured in light blue, Hsp90B in dark blue, XAP2 in orange and AHR in green. Nucleotide molecule, molybdate ion and indirubin ligand are shown in sticks.

File name: Supplementary Movie 2

Description: Close-up view showing PAS-B domain and its interaction with Hsp90 and XAP2. The indirubin molecule is shown as sticks and coloured in magenta.

File name: Supplementary Movie 3

Description: 360°-view of the indirubin binding site. AHR residues interacting with the ligand are shown as magenta sticks and labelled. Hydrogen bonds are indicated as dashed blue lines.
