# Supplementary material for: Cryo-EM structure of the agonist-bound Hsp90-XAP2-AHR cytosolic complex
Source: Nat Commun. 2022 Nov 16;13:7010. doi: 10.1038/s41467-022-34773-w (PMC9668932; doi:10.1038/s41467-022-34773-w)
Supplement: Supplementary file 7 — Reporting Summary [file 41467_2022_34773_MOESM7_ESM.pdf]

Corresponding author(s): Jakub Gruszczyk & William Bourguet

Last updated by author(s): Nov 1, 2022

Reporting Summary

Nature Portfolio wishes to improve the reproducibility of the work that we publish. This form provides structure for consistency and transparency in reporting. For further information on Nature Portfolio policies, see our Editorial Policies and the Editorial Policy Checklist.

Statistics

For all statistical analyses, confirm that the following items are present in the figure legend, table legend, main text, or Methods section.

n/a	Confirmed	
☐	☒	The exact sample size (*n*) for each experimental group/condition, given as a discrete number and unit of measurement
☐	☒	A statement on whether measurements were taken from distinct samples or whether the same sample was measured repeatedly
☒	☐	The statistical test(s) used AND whether they are one- or two-sided<br>*Only common tests should be described solely by name; describe more complex techniques in the Methods section.*
☒	☐	A description of all covariates tested
☒	☐	A description of any assumptions or corrections, such as tests of normality and adjustment for multiple comparisons
☐	☒	A full description of the statistical parameters including central tendency (e.g. means) or other basic estimates (e.g. regression coefficient) AND variation (e.g. standard deviation) or associated estimates of uncertainty (e.g. confidence intervals)
☒	☐	For null hypothesis testing, the test statistic (e.g. *F*, *t*, *r*) with confidence intervals, effect sizes, degrees of freedom and *P* value noted<br>*Give P values as exact values whenever suitable.*
☒	☐	For Bayesian analysis, information on the choice of priors and Markov chain Monte Carlo settings
☒	☐	For hierarchical and complex designs, identification of the appropriate level for tests and full reporting of outcomes
☒	☐	Estimates of effect sizes (e.g. Cohen's *d*, Pearson's *r*), indicating how they were calculated

Our web collection on statistics for biologists contains articles on many of the points above.

Software and code

Policy information about availability of computer code

Data collection	Cryo-EM data collection was performed using Titan Krios transmission electron microscope (Thermo Fisher Scientific) operating at 300 kV and equipped with Gatan K3 direct electron detector camera (Gatan) and GIF Quantum energy filter (Gatan) set to a slit width of 20 eV. Automated data collection was done using EPU v.2.11 software.
Data analysis	Cryo-EM data were processed using RELION v.3.0 and 3.1, MotionCor v.2, CTFFIND v.4.1, Chimera v.1.13.1 and ChimeraX v.1.3. Model building was done using Chimera v.1.13.1, ChimeraX v.1.3, Coot v.0.8.9.2, I-Tasser (https://zhanggroup.org/I-TASSER/) and Grade (http://grade.globalphasing.org). Figures were generated using UCSF Chimera v.1.13.1 and ChimeraX v.1.3. Ligand binding data were analyzed with GraphPad Software v.5.0 Inc., San Diego, CA. PAS-B topology diagram was obtained using Pro-origami (http://munk.cis.unimelb.edu.au/pro-origami/). The cavity within the PAS-B domain was calculated using CASTp (http://sts.bioe.uic.edu/castp/calculation.html). Sequence alignment was performed using Clustal Omega (https://www.ebi.ac.uk/Tools/msa/clustalo/) and visualized using ESPript v.3.0 (https://espript.ibcp.fr). Plots were generated using Prism v.9 (GraphPad). MS data were processed using MassLynx v.4.2.<br>For MD simulations, the following software was used: Gromacs v.2022, AmberTools v.20 and UCSF Chimera: v. 1.14. Force field parameters ff19sb, OPC and GAFF2 as well as algorithms v-rescale, Parrinello-Rahman, LINCS and Particle Mesh Ewald were implementations within the Gromacs v.2022 and/or AmberTools v.20. Gmx programs rmsf, gangle, pairdist were part of Gromacs v.2022.

For manuscripts utilizing custom algorithms or software that are central to the research but not yet described in published literature, software must be made available to editors and reviewers. We strongly encourage code deposition in a community repository (e.g. GitHub). See the Nature Portfolio guidelines for submitting code & software for further information.

Data

Policy information about availability of data

All manuscripts must include a data availability statement. This statement should provide the following information, where applicable:

- Accession codes, unique identifiers, or web links for publicly available datasets
- A description of any restrictions on data availability
- For clinical datasets or third party data, please ensure that the statement adheres to our policy

The atomic coordinates for the indirubin-bound Hsp90-XAP2-AHR complex have been deposited in the Protein Data Bank (PDB) under the accession code 7ZUB. The cryo-EM map obtained in this study has been deposited in the Electron Microscopy Data Bank (EMDB) under the accession code EMD-14971. Focused map used for model refinement has been deposited in EMDB under the accession code EMD-14972.
Protein sequences used in this study: human aryl hydrocarbon receptor (AHR, UniProt accession number P35869), heat shock protein HSP 90-beta (Hsp90, UniProt accession number P08238), HBV X-associated protein 2/aryl-hydrocarbon receptor-interacting protein (XAP2/AIP, UniProt accession number O00170) and prostaglandin E synthase 3 (p23, UniProt accession number Q15185).
Atomic models used in model building: 5FWP for Hsp90, 2LKN and 4AIF for XAP2, 4F3L and 4ZPR for AHR.

Human research participants

Policy information about studies involving human research participants and Sex and Gender in Research.

Reporting on sex and gender	Not Applicable.
Population characteristics	Not applicable.
Recruitment	Not applicable.
Ethics oversight	Not applicable.

Note that full information on the approval of the study protocol must also be provided in the manuscript.

Field-specific reporting

Please select the one below that is the best fit for your research. If you are not sure, read the appropriate sections before making your selection.

☒ Life sciences ☐ Behavioural & social sciences ☐ Ecological, evolutionary & environmental sciences

For a reference copy of the document with all sections, see nature.com/documents/nr-reporting-summary-flat.pdf

Life sciences study design

All studies must disclose on these points even when the disclosure is negative.

Sample size	No statistical methods were used to predetermine sample size. The amount of protein sample used for the biochemical experiments was chosen based on the previous experience for the particular experiment and commonly used sample size in the field of research. For single particle reconstruction, sample size was determined by available microscope time and the number of particles on each micrograph obtained during data collection. 9,300 movies in total were collected for the sample of the Hsp90-XAP2-AHR complex. All cryo-EM maps generated from the movies were sufficient for atomic model building and structure refinement.
Data exclusions	No data was systematically excluded. The procedure of generating cryo-EM maps involves sorting out the particles that are false-positively picked, damaged or represent minor conformations. The procedure is part of the RELION v.3.0 and 3.1 pipeline.
Replication	The number of the replications is stated in the Figure Legends.
Randomization	The experiments were not randomized as this study did not allocate experimental groups.
Blinding	Blinding was not required for this study because no subjective allocation was involved.

Reporting for specific materials, systems and methods

We require information from authors about some types of materials, experimental systems and methods used in many studies. Here, indicate whether each material, system or method listed is relevant to your study. If you are not sure if a list item applies to your research, read the appropriate section before selecting a response.

Materials & experimental systems

n/a	Involved in the study
☐	☒ Antibodies
☐	☒ Eukaryotic cell lines
☒	☐ Palaeontology and archaeology
☒	☐ Animals and other organisms
☒	☐ Clinical data
☒	☐ Dual use research of concern

Methods

n/a	Involved in the study
☒	☐ ChIP-seq
☒	☐ Flow cytometry
☒	☐ MRI-based neuroimaging

Antibodies

Antibodies used

The primary antibodies used in this study: rat anti-MBP, Sigma-Aldrich, dilution 1:1,000, catalogue number SAB4200082-200UUL, lot number 039M4753V, rat anti-DYKDDDDK (anti-FLAG), BioLegend, dilution 1:1,000, clone L5 catalogue number 637301, lot number B318853, mouse anti-beta-actin, Proteintech, dilution 1:5,000, catalogue number 66009-1iG, lot number 10004156, rat anti-Myc-tag, ABCAM, dilution 1:1,000, clone number [9E10], catalogue number ab206486, lot number GR3412503-4, rat anti-Strep-tag, ABCAM, dilution 1:2,000, clone number [11A7], catalogue number ab252885, lot number GR3352502-03. The secondary antibodies used in this study: sheep anti-mouse HRP conjugated, GE Healthcare, dilution 1:3,000, catalogue number GENA931-1ML, lot number 16982037 and goat anti-rat HRP conjugated, BioLegend, dilution 1:5,000, clone number poly4054, catalogue number 405405, lot number B321857.

Validation

The anti-MBP antibody was validated using Western blotting as described on the product's web site (https://www.sigmaaldrich.com/FR/en/product/sigma/sab4200082). The anti-DYKDDDDK antibody was validated using Western blotting as described on the product's web site (https://www.biolegend.com/en-us/products/purified-anti-dykddddk-tag-antibody-4905?Clone=L5). The anti-beta-actin antibody was validated using Western blotting as described on the product's web site (https://www.ptglab.com/products/Pan-Actin-Antibody-66009-1-Ig.htm). The anti-Myc-tag antibody was validated using Western blotting as described on the product's web site (https://www.abcam.com/myc-tag-antibody-9e10-ab206486.html?productWallTab=Abreviews). The anti-Strep-tag antibody was validated using Western blotting as described on the product's web site (https://www.abcam.com/strep-tag-ii-antibody-11a7-ab252885.html). The goat anti-mouse antibody was validated using Western blotting as described on the product's web site (https://www.sigmaaldrich.com/FR/en/product/sigma/gena9311ml). The goat anti-rat antibody was validated using Western blotting as described on the product's web site (https://www.biolegend.com/fr-fr/products/hrp-goat-anti-rat-igg-minimal-x-reactivity-1396?Clone=Poly4054).

Eukaryotic cell lines

Policy information about cell lines and Sex and Gender in Research

Cell line source(s)

All cell lines used in this study were obtained from commercial sources: Sf9 (Oxford Expression Technologies Ltd), HEK293F (Thermo Fisher Scientific). The human epithelial HeLa-S3, the zebrafish liver ZFL and the rat hepatoma H-4-II-E cells were provided from ATCC (France distributor LGC Standards, Molsheim, France).

Authentication

No authentication was performed as the cell lines were obtained from commercial sources.

Mycoplasma contamination

The cell lines were not tested form mycoplasma contamination.

Commonly misidentified lines
(See ICLAC register)

We did not use the commonly misidentified cell lines in this study.
